# Intervention in professional dance students to increase mental health- and nutrition literacy: A controlled trial with follow up

**DOI:** 10.3389/fspor.2022.727048

**Published:** 2022-09-21

**Authors:** Therese Fostervold Mathisen, Christine Sundgot-Borgen, Beate Anstensrud, Jorunn Sundgot-Borgen

**Affiliations:** ^1^Department of Health, Welfare and Organisation, Østfold University College, Fredrikstad, Norway; ^2^Division of Mental Health and Addiction, Regional Department for Eating Disorders, Oslo University Hospital, Oslo, Norway; ^3^The Norwegian Association of Youth Mental Health, Oslo, Norway; ^4^Institute of Sports Medicine, Norwegian School of Sport Sciences, Oslo, Norway

**Keywords:** body appreciation, eating disorders, energy availability, injuries (MeSH), sports nutrition, mental health, health literacy

## Abstract

**Introduction:**

There is a need to change the culture within the art of dance, as it has been associated with injuries and mental health consequences. This study evaluates an intervention designed to increase mental health literacy, enhance nutritional knowledge, reduce symptoms and effects of low energy availability, and strengthen understanding of sports nutrition and recovery strategies, in dance students of mixed genders.

**Material and methods:**

A total of 125 dance students received three workshops, with 39 arts and crafts students serving as references. The results were evaluated by the Eating Disorder Examination questionnaire, the Low Energy Availability in Females questionnaire, the Hopkins Symptom Check List, and questions on mental health literacy, sports nutrition, and recovery knowledge.

**Results:**

Dance students achieved sustained improvements in mental health and nutrition knowledge and temporary improvements in driven exercise (i.e., performing exercise because of a compulsive drive). No other benefits were identified from the intervention.

**Conclusion:**

Our findings indicate the need for an ongoing education program to reduce the occurrence and development of negative mental health outcomes and low energy availability in professional dance students. Such approaches may not only improve the mental health of dance students but also potentially prevent the high frequency of injuries.

## Introduction

Athletes who participate in sports that emphasize body appearance and thinness/leanness have an increased risk for mental health challenges such as the development of anxiety, depression, and eating disorders (EDs), in addition to low energy availability (LEA) [1–4]. LEA may result from challenges with one's mental health, be the cause of the development of negative mental health symptoms, or even exacerbate existing mental health challenges [2]. LEA causes the symptom spectrum called Relative Energy Deficiency in Sport (RED-S), which is associated with a multitude of clinical consequences whether physical, mental, and/or performance impairments following the mismatch between energy intake and needs [2].

The traditions within dance emphasize an idealization of the lean physique, with performance evaluated subjectively either individually, in pairs, or as groups [3, 5]. Additionally, dance is considered a weight-sensitive sport as the dancers rely on the ability to withstand repeated jumps, lifts, and landings. These features of the sport, being both body image conscientious and physically demanding, often result in a high incidence of mental health complications, LEA, and physical injuries in professional dancers [3, 6–8]. Nevertheless, these findings suggest that dancers possess an insufficient literacy of mental health [6, 9, 10] (i.e., the ability to gain access to, understand, and use information in ways which promote and maintain good health) [11], which could be related to the low priority psychology course included within the university curriculum of collegiate dancers [10]. With the experience of cultural- and/or self-stigma [12], low mental health literacy may hinder dancers from either accessing needed primary healthcare services or receiving optimal treatment [13]. To reduce the effects of mental health challenges and LEA, increased awareness [6, 7, 9, 14, 15], educational programs [3, 6, 7, 14, 16, 17], and reporting effects from program implementation [3, 6, 7, 14, 16–18] are encouraged.

While body dissatisfaction correlates with impairment of mental health and increased risk for eating disorders [1, 19], body appreciation (i.e., accepting and respecting one's body, holding favorable opinions toward it; including its functional capabilities) [20] facilitates a health-promoting behavior and the resistance to sociocultural trends or body idealization [20]. While a previous study on students within exercise science highlighted the importance of body appreciation to withstand body appearance pressure and thinness idealization [21], we recently identified professional dance students of the same nationality and age range to present with mediocre scores in body appreciation [22]. This is particularly concerning, as body appreciation was found to explain the variability in symptoms of eating disorders (i.e., a high level of body appreciation associated with low symptoms of eating disorders) [22].

Currently, educational and preventive programs addressing mental health and nutritional needs in athletes and dancers are scarce. Three studies have been successful at reducing body figure idealization, symptoms of eating disorders, and/or new cases of EDs in young athletes and ballet dancers [23–25]. One of these, a non-controlled study, identified a reduction in symptoms of EDs in adolescent dancers for 3 years, after systematic changes in the dance school environment and repeated peer discussion on body figure idealization [25]. Another study, being a randomized controlled trial design, found a reduction in new cases of EDs in female adolescent athletes in elite sports in high schools by focusing on increasing self-esteem and knowledge about nutrition and psychology, through systemic changes and repeated lectures spanning 1 year [24]. Finally, the most recent randomized controlled trial achieved a reduction in symptoms of EDs and in the idealization of the thin body figure after three group-based discussions aimed at challenging unhealthy body figure idealization [23]. Additionally, two other studies showed an improvement in nutritional knowledge and somatic health in young female, amenorrhoeic athletes and ballet dancers [26, 27]. Furthermore, the university experience can encompass a time when specific mental and nutritional challenges can occur to a student [28, 29]. Most of the previous studies mentioned have either targeted adolescents at a pre-professional level or omitted the inclusion of a reference or control group for comparison. As a result, there is little knowledge on the efficacy of prevention programs targeting professional dance students at the university level.

Current knowledge points to the notable effects of interactive interventions compared to didactic techniques, when we aim to improve mental health and specifically reduce the risk for body figure idealization and eating disorders [30–32]. The effectiveness of interactive learning is known from the field of student learning, in which students' active learning techniques result in better learning outcomes than didactic techniques. Interactive health interventions primarily demonstrated positive effects when they focus on risk factors (e.g., body dissatisfaction), open discussions, recruit targeted samples (e.g., those with increased risk profile), contain multiple sessions rather than a single session, and rely on the use of professional competence [30–32]. The dissonance theory proposes a change in personal thought processes when differences or conflicts arise between thoughts, knowledge, and/or actual behavior [33]. This may explain the optimal intervention outcomes achieved from interactive, dissonance-based interventions that induce this conflict between behaviors and thought processes [31, 32]. One could therefore hypothesize that improvement in health behavior could occur if knowledge is increased, and cultural and individual actions, as well as behavior, are interactively addressed and discussed in selected groups.

The objective of this study was to evaluate a short intervention of three separate interactive workshops, designed to increase mental health literacy and to improve knowledge of and actions related to LEA, sports nutrition, and recovery strategies, in professional dance students of mixed genders. To further develop our understanding of the possible effects of these interventions, this study included a reference group for comparison purposes. The intervention design was built on previously identified effective elements (i.e., targeting a selected group, including interactive elements, bringing in professional competence, and increasing engagement and learning by relying on a multisession design) [24, 30, 34]. We hypothesized that (1) professional dance students have lower mental health literacy compared to a non-athletic reference group; (2) the intervention would increase mental health literacy, (3) the intervention would increase body appreciation and reduce self-reported symptoms of mental and physical health challenges, and finally; (4) that the intervention would increase knowledge related to sports nutrition and recovery needs.

## Methods

### Design

**Figure 1:**
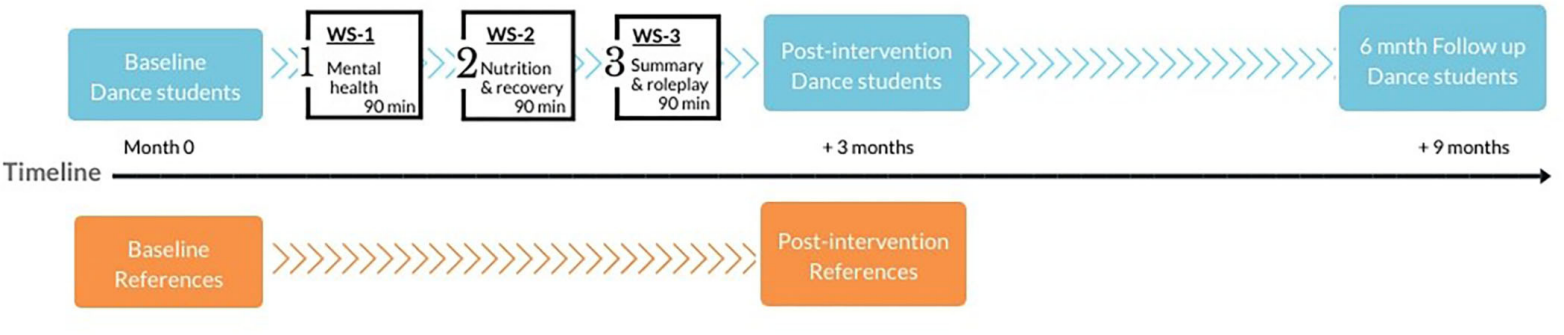
A diagram illustrating the flow of interventions and evaluations. Blue/dark and orange/light boxes illustrate the time for evaluations of dance students and references, respectively, and white boxes illustrate workshop interventions for dance students and their main focus. WS, workshop.

This was a longitudinal study with a parallel group design, evaluating the effects of intervention for dance students and comparing outcomes to a reference group. The intervention was held during regular school hours during the fall semester of 2019. Effects were measured with electronic questionnaires distributed at baseline, post-intervention, and 6 month follow-up (Figure 1).

### Recruitment

Recruitment of dance students and references were targeted by affiliation and/or study specialization. The dance students recruited included all students at the two national universities for dance professionalization in Norway. After obtaining consent from the academic deans at the two national dance schools, students and teachers were informed about the study through a meeting and an e-mail with an individual request for participation.

The reference group was recruited using the same procedure as conducted with the dance students and chosen based on the assertion of a comparable expectation of performance pressure, but with less focus on the body, appearance, and physical fitness (i.e., arts and crafts artists and actors). As such, these students represented ideal comparatives regarding the performance aspect (which may cause mental health challenges) but without the expected issues from specific body figure idealization. Due to difficulty in recruitment of references, this group was offered a gift certificate of €24 if they completed the questionnaire at baseline and post-intervention. Because of this challenge in recruitment and retention of the reference group, data were obtained only at baseline and post-intervention and not at follow-up.

### Participants

A total of 141 dance students were invited to participate, from which 125 were included in this study (see Table 1), with the remaining excluded due to lack of response. The dance students represented jazz dance (*n* = 72) and contemporary dance (*n* = 53) and were in either the 1st (*n* = 46), 2nd (*n* = 38), or 3rd (*n* = 41) year of a bachelor's degree program. A total of 202 reference students were invited, from which 39 responded and were included in the study (see Table 1). The reference students studied arts and crafts (*n* = 18), theater (*n* = 17), or scenography and performance art (*n* = 4).

**Table 1:** Baseline demographics of participants; values are mean (99% CI) and comparisons are made between groups and separately for sexes between the two groups.

**School**	**Age, years**	**BTW diff,** ***p*-value**	**BMI, kg × m^−2^**	**BTW diff,** ***p*-value**	**Exercise, h/wk**	**BTW diff,** ***p*-value**
Intervention, total	20.7 (20.3, 21.1)		21.4 (20.9, 21.9)		26.4 (23.5, 29.3)	
Dance Men (11%)	21.5 (19.8, 23.2)		20.9 (19.6, 22.3)		30.1 (23.6, 36.7)	
Dance Women (89%)	20.6 (20.1, 20.9)		21.5 (20.9, 22.0)		25.9 (23.0, 28.8)	
References, total	28.7 (25.1, 32.3)	7.6 (6.1, 9.0) *p* < 0.001	23.3 (21.9, 24.7)	1.9 (1.0, 2.8) *p* < 0.001	9.2 (4.2, 14.1)	17.2 (11.6, 22.8) *p* < 0.001
Reference Men (15%)	27.8 (15.9, 39.7)	7.5 (2.9, 12.1) *p* = 0.003	22.3 (15.1, 29.4)	-	12.3 (2.4, 22.3)	17.8 (5.9, 29.7) *p* < 0.001
Reference Women (85%)	28.8 (24.7, 32.9)	7.5 (6.0, 9.1) *p* < 0.001	23.5 (22.1, 24.9)	2.0 (1.1, 3.0) *p* = 0.001	8.5 (3.0, 14.1)	17.3 (11.1, 23.7) *p* < 0.001

BTW, between-groups; Diff, difference between groups; BMI, body mass index; h, hours; wk, week.

### Intervention

The intervention consisted of three 90-min workshops covering different health topics and was held during school hours, one session per month for 3 consecutive months. The content of the intervention was linked with a mandatory curriculum in several school subjects, which justified the intervention as compulsory for students. Experienced researchers (with expertise in mental health and specifically eating disorders, nutrition, LEA and RED-s, in ordinary school and sports school intervention programs, and in dance specifically), user representatives from the dance environment, dance pedagogues, and members from the volunteer organization The Norwegian Association of Youth Mental Health met to plan the content of the intervention. One health professional (registered dietitian) and one professor in exercise science and health (specialized in prevention and treatment of eating disorders) held the three workshops, each lasting 90 min. Interventions were delivered as planned in the project group (confirmed by the instructors, also being part of the project group). The intervention relied on interactive communication, followed by the best practice suggested (i.e., delivered to a selected/delineated group and by a health professional), and was held as an interactive intervention (i.e., lecture presentation including calls for discussions and role play) and a multisession intervention, which emphasized a focus on body acceptance [24, 30, 34]. In overview, the first workshop addressed physical and mental health (definition, understanding, symptoms, frequency) and performance effects, the second workshop focused on nutrition, recovery strategies, health, and performance (covering the aspects of RED-s), and the third workshop summarized the intervention and demonstrated the content by discussion of case histories (for details on workshops, see Supplementary Table 1).

### Questionnaires

The questionnaire was created by the web-based system SurveyXact 8.2 (Rambøll Management Consulting). Due to time considerations for the references, their questionnaire was shorter in length compared to the questionnaire given to the dance students. To evaluate the outcomes related to hypotheses 1 and 2 (mental health literacy), and given the lack of existing validated questionnaires [35], questions to reflect mental health literacy were created by the project group (the authors, user representatives from The Norwegian Association of Youth Mental Health, and a group of four student representatives and two dance instructors). Evaluation of outcomes related to hypothesis 3 (symptoms of mental and physical health) was done by four validated questionnaires (the Body Appreciation Scale, the Hopkins Symptom Check list, the Eating Disorder Examination Questionnaire for mental health symptoms, and the Low Energy Availability in Females Questionnaire or the Low Energy Availability in Males Questionnaire for physical health symptoms). To evaluate outcomes related to hypothesis 4 (knowledge of nutrition and recovery strategies), the project group created specific questions to measure knowledge of sports nutrition and in recovery strategies.

#### Demographics

Demographic information included gender, age, weight, height, study program, academic year, and exercise volume (hours of dance training at school, in shows, and in other non-dance-specific training).

#### Mental health literacy

The participants were given four questions intended to evaluate mental health literacy, being scored with points ranging from 0 to 3 per question (see Supplementary Table 2). Examples of questions were “When does an everyday problem become a mental health symptom” and “Give 1–3 examples of psychological symptoms on mental health issues (see Supplementary Table 2 for details). Groups' mean scores at baseline, post-intervention, and 6 months follow-up were compared to evaluate the effect of the intervention.

#### Mental and physical health symptoms

##### Body appreciation scale (BAS-2)

The BAS-2 (Cronbach's α = 0.94) consists of 10 items measuring body appreciation with optimal validity and reliability [36]. The items are ranged on a 5-point Likert scale with a higher score indicating a higher level of body appreciation. Examples of items included are “I respect my body,” “I am attentive to my body needs,” and “I am comfortable in my body.”

##### Hopkins symptom checklist (SCL-10)

The SCL-10 (Cronbach's α = 0.88) measures symptoms of depression and anxiety [37]. All 10 questions (depression: six items, anxiety: four items) are scored on a 4-point Likert scale, where a higher score indicates greater severity of symptoms. The scores are summarized into a global score, with a cutoff for mental health issues at 1.85. Examples of items included are “During the current week, have you felt suddenly scared for no reason?”, “During the current week, have you had sleeping difficulties?”, and “During the current week, have you felt that everything is an effort?”.

##### Low energy availability in females questionnaire (LEAF-Q)

The LEAF-Q screens for LEA in female athletes and identifies the occurrence of injuries, gastrointestinal dysfunction (GD), and menstrual irregularities (MI) [38]. It has optimal sensitivity and specificity in female endurance athletes and dance students [38]. Suggested cutoff's for GD, MI, and the total LEAF-Q scores are ≥2, ≥4, and ≥8, respectively, with a higher score indicating a more severe clinical condition. Each question contained pre-determined responses, which is coded based on an evaluation of severity. An example of a question from the subscale injuries section included “Have you been injured during the current year, depriving you from the usual training- or competition participation?”. Similarly, an example of one of the questions from the gastrointestinal function subscale is “Do you have cramps or abdominal pain that are not due to the menstrual cycle?”. Finally, examples of questions taken from the menstrual irregularity subscale included “Do you use hormonal contraceptives?” and “What are the reason for using hormonal contraceptives?”.

##### Low energy availability in males questionnaire (LEAM-Q)

The LEAM-q measures symptoms of low energy availability in men, which originally contained five subscales (dizziness; gastrointestinal function; temperature regulation in rest; injury and illness; wellbeing and recovery) covering 33 items (included in this study). A multinational validation study, encompassing men from different sports, identified four items containing sex drive in the subscale of wellbeing and recovery that presented with the strongest sensitivity and specificity in identifying those at risk for LEA [39]. We, therefore, included only these four items in our results in addition to the information provided on injury rate and severity. The items are scored according to clinical severity and grouped into either low or normal libido, which serves as symptoms of LEA [39]. Examples of sex-drive questions included “In general I would rate my sex drive as [choose from 4 levels]” and “Over the past month, I have woken up in the morning with erection [chose from 4 grouped frequencies].”

##### Eating disorder examination questionnaire (EDE-q)

The EDE-q (Cronbach's α = 0.95) consists of 28 items, which are measuring symptoms of eating disorders [40]. A global score is calculated from 22 of the total items and scored on a 7-point Likert scale. Examples of questions included “Have you been deliberately trying to limit the amount of food you eat to influence your shape or weight (whether or not you have succeeded)?” and “Have you had a definite fear that you might gain weight?.” The remaining six items measure the frequency of binge eating and compensatory behavior over the last 28 days. In women, a global score above 2.5 indicates a high probability of having an eating disorder [41]. In addition, the occurrence of binge eating or compensatory behavior ≥ four episodes per month is considered above the clinical cutoff for the diagnosis of an eating disorder.

#### Knowledge in sports nutrition

The participants were given questions about nutrition to evaluate their knowledge of foods as sources of nutrients and that are considered of importance to athletes. Examples of open-ended questions included “List four food items that are good sources of carbohydrate” and “List four food items that are good sources of healthy fat” (see Supplementary Table 3 for scoring). Scores of 0 to 4 were given for each food listed as a source of macronutrient, and scores of 0 to 3 were marked for each food listed as a source of a micronutrient. Groups' mean scores at baseline, post-intervention, and 6 month follow-up were compared to evaluate the effect of the intervention.

#### Knowledge on recovery strategies

The participants were given a question on knowledge of recovery strategies, in which they could list their personal recovery measures (specifically saying: “Which recovery measures do you comply to during your typical week with training?”). All answers were scored with a range of 0 to 4 points, with one point for each of the following: sleep, alternative low-intensity activities, food intake, or ≥1 day free for training. Additionally, as energy intake is typically associated with injuries and LEA in weight-sensitive sports, the frequency of students mentioning food intake as a recovery strategy was separately registered.

#### Evaluation of workshop content

The post-intervention questionnaire asked the dance students to rate their experience with the workshop, on a Likert scale from 1 (very useful) to 5 (not useful) with two additional choices of [6] “I don't remember” and [7] “I was not present.” The questions were specifically asked about their experience with intervention content/focus and how they rated their post-intervention knowledge on mental health, sports nutrition, and recovery. Additionally, the dance students were provided with opportunities to present suggestions for improvements with an open-ended question.

#### COVID-19 effect on life and perceived mastery of life challenges

Because the 6 month follow-up measure coincided with the onset of the COVID-19 pandemic, we included questions about how the dance students perceived the societal restrictions affected their mental health and responses to the questions on the questionnaire. Answers to the question “Do you think COVID-19 affects your response to this questionnaire” were given by the Likert scale of 1 (no impact) to 6 (very much), and an open-ended follow-up question gave the opportunity to explain further in detail.

### Ethics and safety procedures

This study was approved by the Norwegian Regional Committee for Medical and Health Research Ethics (ID 11472), registered in the Norwegian Center for Research Data (ID 194211) and in a clinical trial (ID: NCT04085861).

Clinical follow-up procedures were implemented by providing a contact person at the dance students' school at the end of the questionnaire, including the health service contacts from the school's official health service program and professional healthcare, if necessary.

### Statistics

All analyses were conducted in SPSS version 26. The linear mixed regression models were built to estimate the between-group differences (dance students vs. references) and the within-group changes (Baseline vs. the two other times). Dependency in the repeated outcome measures was accounted for by including a random intercept factor. The fixed factors were as follows: Group (dance, reference), Time (baseline, post-intervention, follow-up), and Group × Time.

No power calculation was performed, as the aim of this health project was to include the total population of professional dance students in Norway, and in an attempt to provide a control to the intervention effect, a reference group was invited as a comparative. Due to the number of tests, differences with p-values ≤ 0.01 were considered significant. A comparable statistical approach was used for the dichotomous outcome variables, replacing the analysis with a generalized linear model using a binominal distribution and logit link function. The outcome data are presented as estimated means with a 99% confidence interval, and standardized Hedge's g effect sizes for continuous data were calculated. Demographic data presented in Table 1 and attrition analyses were analyzed separately with the independent t-test, the Mann–Whitney U test, or the chi-square analyses as appropriate, with a significance level of a *p* < 0.05.

## Results

In total, 125 dance students [111 (89%) women and 14 (11%) men] and 39 references (33 (85%) women and 6 (15%) men) participated at baseline (Table 1). At post-intervention, there were 71 dance students and 29 references participating, with a rate of missing corresponding to 43 and 26% respectively. The same number of dance students responded to the 6 month-follow-up questionnaire.

Attrition analyses in dance students revealed only the academic year to be different, with significantly more missing among 3rd-year students compared to 2nd-year students (*p* = 0.04). Among references, only current age (*p* = 0.002), age when their art/performance interest was initiated (*p* = 0.019), and symptoms of depression and anxiety (*p* = 0.03) differed between responders and dropouts. Attrition analyses for dance students at 6 month follow-up found no differences between responders (*n* = 71, 56%) and dropouts (*n* = 57, 45%) from the post-intervention tests.

The dancers had a significantly higher volume of exercise vs. references at all times, and additionally, the dancers increased their volume at 6 month follow-up by an estimated mean (CI 99%) of 3.8 (0.6, 7.5) h/week, *p* = 0.009.

### Mental health literacy

**Figure 2:**
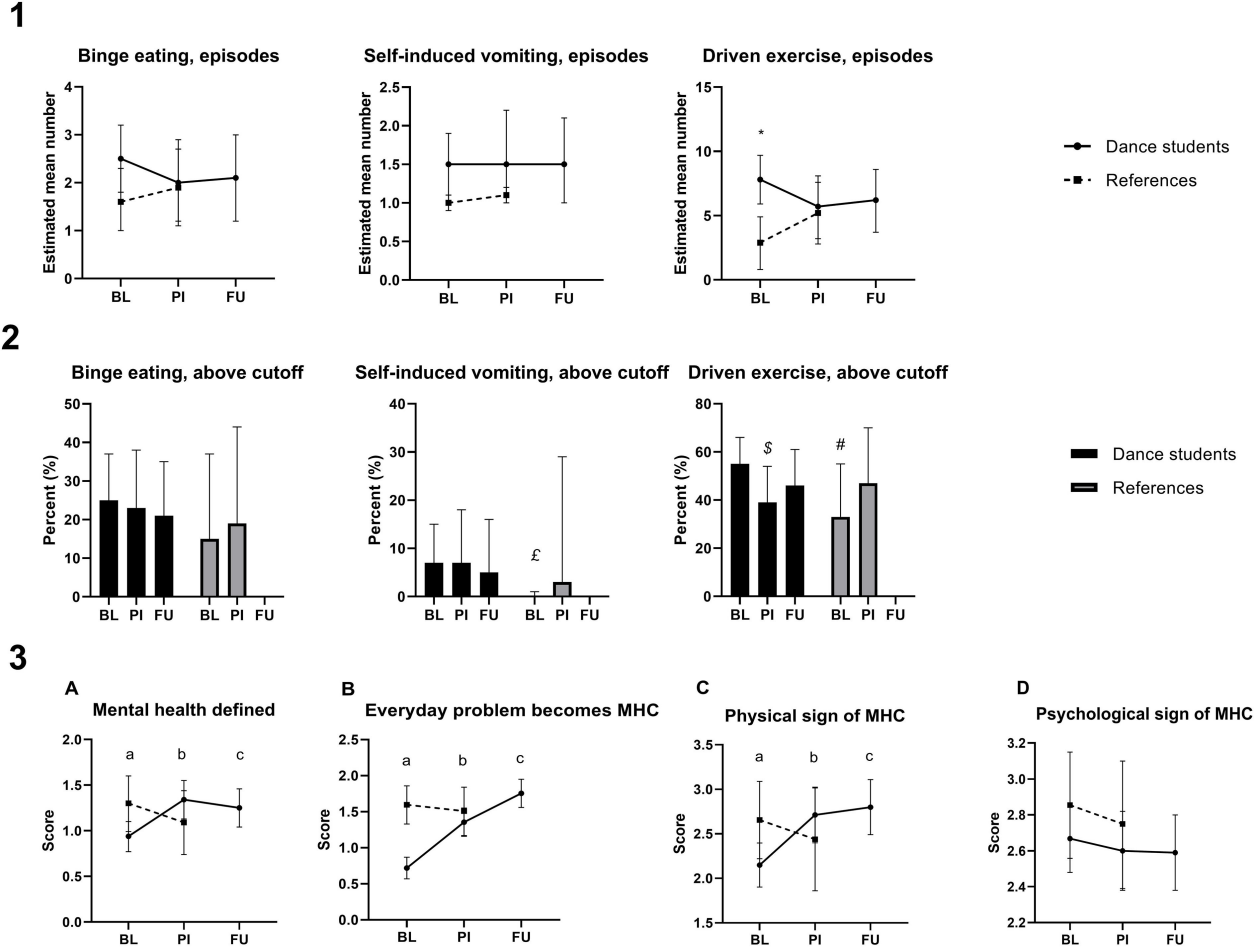
Health behavior and health literacy. The estimated mean (CI 99%) number of episodes with binge-eating and purging among dance students and references across time (Row 1), and the percentage above the respective clinical cutoffs (Row 2). Row 3 illustrates results from mental health literacy questions: “What is mental health to you?” **(A)**, “When does an everyday problem become a mental health challenge?” **(B)**, “Mention three physical signs of mental health challenges” **(C)**, and “Mention three psychological signs of mental health challenges” **(D)**. BL, baseline; PI, post-intervention; FU, 6 months follow-up; MHC, mental health challenge; *significant between-group difference (mean (CI 99%) differences of 4.9 (1.3, 8.6), g = 0.64, *p* = 0.001; £significant between-group difference (mean (CI 99%) differences of 6.9 (1.3, 12.3), g = 4.09, *p* = 0.002;^$^ significant within-group change (mean (CI 99%) change of 15.8 (−0.5, 32.0), g = 1.77, *p* = 0.016; ^#^significant between-group difference (mean (CI 99%) differences of 21.4 (−1.5, 44.4), g = 12.9, *p* = 0.016. ^a^significant difference between groups; ^b^significant change from BI among dance students; ^c^significant change from BI among dance students.

Dancers had significantly lower mental health literacy in three of four domains compared to references at baseline (define mental health, *p* = 0.007; when does everyday challenge become a mental health problem, *p* < 0.000; a physical sign of mental health problem, *p* = 0.009; a psychological sign of mental health problem, *p* = 0.16), but improved significantly after intervention, resulting in no between-group difference (*p* > 0.12). The improved literacy was sustained at follow-up among the dancers (Figure 2, row 3).

### Mental and physical health symptoms

Estimated mean scores for outcomes related to mental health (BAS-2, SCL-10, and EDE-q) are presented in Table 2. Frequency of binge eating and purging behavior, being symptoms of disordered eating measured by the EDE-q, in dance students and references are illustrated in Figure 2. While dancers as a group had numerically more episodes of binge-eating and purging on average at baseline, only the frequency of driven exercise was different from references (Figure 2, row 1). Fluctuations by time within each group resulted in no further differences at the two subsequent measurement times. The numbers of individual dancers with episodes of self-induced vomiting above the clinical cutoff at baseline were significantly higher compared to references, as were the numbers with episodes of driven exercise (Figure 2, row 2).

**Table 2:** Symptoms of mental health challenges, mental health literacy, and knowledge in sports nutrition and recovery strategies.

**Mental health**					
	**Baseline**	**Post**	**Within group Δ, g, *p***	**FU**	**Within group Δ, g, *p***
**BAS-2**					
Dance student	3.5 (3.3, 3.7)	3.5 (3.3, 3.7)	3.7 (3.4, 3.9)	
References	3.8 (3.4, 4.1)	3.8 (3.4, 4.2)	-	
**SCL-10** Dance students % ≥cutoff References % ≥cutoff	2.1 (1.9, 2.2) 55 (43, 66) 1.8 (1.5, 2.1) 45 (26, 66)	2.2 (2.0, 2.4) 60 (45, 73) 1.9 (1.5, 2.2) 41 (21,65)		1.9 (1.7, 2.1) 48 (34, 62) - -	
**EDE-q global** Dance students % ≥cutoff References % ≥cutoff	1.5 (1.3, 1.8) 21 (13, 32) 1.2 (0.7, 1.7) 13 (4, 35)	1.3 (1.0, 1.6) 13 (6, 26) 1.2 (0.6, 1.7) 10 (2, 35)		1.3 (1.0, 1.6) 17 (8, 30) - -	
**Knowledge of sports nutrition**					
	**Baseline**	**Post**	**Within group** **Δ, g**, ***p***	**FU**	**Within group** **Δ, g**, ***p***
**Carbohydrate**					
Dance students References	3.0 (2.8, 3.3) 2.8 (2.4, 3.3)	3.3 (3.0, 3.6)* 2.9 (2.4, 3.4)	0.3 (0.0, 0.6) g = −0.26 *p = 0.01*	3.3 (3.0, 3.6) -	0.3 (0.0, 0.6) *g* = −0.36 *p = 0.006*
**Protein**					
Dance students References	2.8 (2.6, 3.1) 2.8 (2.3, 3.3)	3.1 (2.7, 3.4) 2.7 (2.2, 3.3)		3.2 (2.9, 3.6)* -	0.4 (0.0, 0.8) *g* = −0.32 *p = 0.005*
**Unsaturated fats**					
Dance students References	2.2 (1.9, 2.5) 2.3 (1.8, 2.8)	2.7 (2.3, 3.0)* 2.4 (1.8, 3.0)	0.5 (0.1, 0.8) g = −0.34 *p < 0.001*	2.9 (2.6, 3.3)* -	0.8 (0.4, 1.1) *g* = −0.54 *p < 0.001*
**Calcium**					
Dance students References	1.7 (1.4, 1.9) 1.8 (1.4, 2.2)	1.8 (1.6, 2.1) 1.8 (1.3, 2.3)		2.2 (1.9, 2.5)* -	0.5 (0.2, 0.8) *g* = −0.57 *p < 0.001*
**Iron**					
Dance students References	(0.9, 1.3) 1.2 (0.8, 1.5)	1.5 (1.2, 1.7)* 1.1 (1.2, 1.7)	0.4 (0.1, 0.6) *g* = −0.56 *p < 0.001*	1.5 (1.2, 1.7)* -	0.4 (0.1, 0.6) *g* = −0.44 *p < 0.001*
**Knowledge on recovery strategies**					
	**Baseline**	**Post**	**Within group** **Δ, g**, ***p***	**FU**	**Within group** **Δ, g**, ***p***
**Recovery, general**					
Dance students References	1.7 (1.5, 1.9) 1.2 (0.8, 1.6)	1.8 (1.5, 2.0) 1.2 (0.8, 1.6)		1.8 (1.6, 2.0) -	
	**BTW diff**. 0.35 (0.12, 0.58), *g* = 0.65 *p < 0.001*	**BTW diff**. 0.33 (0.1, 0.6), *g* = 1.25 *p < 0.002*			
**Recovery, diet** (%)					
Dance students References	60 (48, 71) 25 (10, 49) **BTW diff**. 35 (12, 58), *p < 0.001*	59 (44, 73) 26 (9, 54) **BTW diff**. 33 (6, 61), *p = 0.002*		68 (53, 80) -	

Baseline, scores before dance-intervention; Post, scores after dance-intervention; FU, 6 months follow up after dance-intervention; h, hours; BAS-2, Body appreciation scale-2; SCL-10, Symptom Check List-10; EDE-q, Eating Disorder Examination questionnaire; Δ, size of within-group change from baseline; g, Hedges g for effect size; BTW diff., between-group difference; ^*^denotes for which group the within-group changes are reported.

Estimated mean (CI 99%) scores in mental health outcomes, knowledge on sports nutrition and recovery strategies in dance students and references, and frequency in each group mentioning diet specifically as a recovery strategy (recovery, diet). Where changes or differences were identified, the mean differences and their calculated effect size are presented.

#### Symptoms of LEA and frequency of injuries

At baseline, the estimated mean (CI 99%) LEAF-Q total score in the group of female dancers was 8.4 (7.4, 9.5), and the numbers above the LEAF-Q cutoff were 57% [45, 69]. The corresponding results for LEAF-Q menstrual irregularities were 3.4 (2.8, 4.0) points and 37% [26, 50] and for LEAF-Q gastrointestinal dysfunction were 3.4 (2.8, 4.0) points and 79% [68, 88]. There were no significant changes over time. In male dancers, the estimated (CI 99%) numbers identified with symptoms of low libido (i.e., symptoms of LEA) were 93% [43, 97] at baseline, with no changes over time.

In total, 53% [41, 65] of the female dancers and 14% [18, 57] of the male dancers reported injuries depriving them of physical practice. There were no statistical changes in these rates over time.

### Knowledge in mental health, sports nutrition, and recovery strategies

The scores for mental health literacy are illustrated in Figure 2, and scores for knowledge in sports nutrition and recovery strategies are presented in Table 2. While knowledge of mental health was lower among dance students compared to references at baseline, dance students improved their knowledge with long-term effect, resulting in no between-group difference at post-intervention and follow-up. Dancers had better knowledge of recovery strategies compared to references at baseline but did not improve further after the intervention (Table 2). While dancers improved their knowledge of nutrition after the intervention, there was no between-group difference in references at any time (Table 2).

### Perceived mastery and quality of life during early COVID-19

In total, 47 dance students (37%) reported that COVID-19 had most likely or very likely affected their response to the 6 month follow-up questionnaire. Among these, four dance students reported to have experienced increased wellness and calmness, while the remaining reported a negative impact, typically being increased anxiety and stress (four persons), increased worries about body weight/figure following increased food intake (two persons), or accompanied by a voluntary decreased food intake (four persons), and worries for a future career (six persons). One reported having decided to stop the dance career.

### Evaluation of intervention workshops

At post-intervention, 73% of dance students rated the intervention as very useful, useful, or somewhat useful, with 23% being neutral and 4% finding no benefits from participating. In total, 57 students (45%) requested more comprehensive information on topics included in the workshops, with 23 of them (18%) requesting more information on individualized nutrition needs, 26 (20%) of them on mental health (e.g., how to identify symptoms of mental health challenges, or how to deal with periods with injuries), and 8 of them (6%) on recovery strategies.

## Discussion

This is the first intervention to address mental health literacy and symptoms and knowledge of sports nutrition and recovery strategies in professional dance students at the university level. We identified initially a lower mental health literacy among dancers vs. the non-active reference group; however, after implementation of the intervention, we noted several improvements in knowledge and behavior, although symptoms of mental and physical health remained unchanged. Although the majority rated the intervention as useful, we were not able to find strong evidence for any sustained effects other than an increase in the knowledge base. Partly supporting the first and second hypotheses was the finding of lower mental health literacy in three of four domains with the dance students compared to the references, as well as an improvement in mental health literacy in dance students after the intervention. Nevertheless, these improvements did not result in any additional advantages compared to references (no difference between groups). In support of our third hypothesis, fewer dance students were above the clinical cutoff in driven exercise post-intervention. However, this effect was not observed at follow-up (i.e., 6 months post-intervention). In comparison to the third hypothesis, the intervention did not result in any improvements in any other mental health outcomes. Partly supporting our fourth hypothesis, the intervention caused a within-group improvement in sports nutrition knowledge; however, the knowledge of dancers was not different from references at any time. In contrast to the last hypothesis, we found no effect of the intervention on recovery strategies.

### Mental health literacy

Our findings support previous reports on low mental health literacy among dancers [6, 13], as they had a reduced knowledge base compared to the references. This short-duration intervention, designed specifically to improve mental health literacy and health aspects important to dancers, was successful at improving mental health literacy. This was an important achievement, as the culture within dance is reported to normalize feelings of pain, strive for perfectionism, and as such potentially teach novices to withstand the feeling of physical and mental exhaustion [5, 42, 43]. The success may lie in the interactive design, in which students were given the opportunity to confront such culture by discussing practical questions and experiences and as such better understand and agree on the boundaries between everyday problems and more serious mental health problems. We previously demonstrated that third-year students in dance were more willing to consult with a professional when experiencing mental health challenges than first- and second-year students [44]. It is reasonable to suggest that, more students, in the earlier stages of their studies and career, may be more willing to consult with professionals when experiencing mental health challenges, after developing an increase in mental health literacy.

### Mental and physical health status

Comparing our findings on mental health and LEA in dance students with other athletic references of similar age, sex, nationality, and period of time [44–46] highlights a concern for the mental and physical health of dance students. As these students have to rely on their mental and physical performance capacity for years to perform as dancers, it is important to find successful interventions to improve or maintain their health at an optimal, sustainable, and healthy level. Unfortunately, we were unable to improve symptoms of LEA, frequency of injuries, or outcomes with mental health. Considering the short time between intervention and evaluation, the lack of improvements in symptoms of mental health challenges and physical health symptoms of LEA may be reasonable. It was previously suggested that any change in mental health requires a longer evaluation period (i.e., >9 months), as some mental health constructs would need time to become established [34]. The same can be deducted about the physical health symptoms of LEA [47]. Participants' age also had a potential impact on the effect. Previous successful interventions on the positive embodiment, quality of life, and prevention of EDs in dancers, athletes, and non-athletes focused on students in high school. Thus, the previous subjects studied were younger compared to the study sample that we evaluated [24–27, 34]. In the current study, the dancers were older and studying toward professionalization in dance, meaning they were becoming more aware of the qualifications required with professional dance positions. As such, it may be difficult to achieve changes and improvements in mental health (i.e., symptoms of body dissatisfaction/low body appreciation, symptoms of disordered eating, and symptoms of mental stress or depression) as the culture within the dance profession has not changed. Additionally, the intensity of intervention exposure and total duration of the project period, specifically when conducted within an environment associated with a long-held negative health practice, may be important to consider. One previous successful study, with a comparable study design (i.e., three 90 min sessions), was probably successful due to its shorter and more intensive intervention period (3 weeks). In addition to the shorter duration of the intervention, this study addressed only one topic (body figure idealization) and included athletes from sports without body figure presentation evaluation [23]. While the single topic design may have allowed for a better chance to directly assess the effectiveness or impact of the dissonance thought processes in a study population, knowledge of the continued existence of a sport-specific body figure idealization and appearance in dance is still a highly relevant explanation for the less favorable results obtained in this study.

Nevertheless, at the university level, previous studies found that the wellbeing of the students is reduced and the levels of anxiety and depression are affected by academic progression. Thus, suggesting being a student at the university level can provide further challenges to mental health [28]. As such, it may also be reasonable to suggest that stabilization of negative mental health behaviors or symptoms in students is a successful outcome. Still, and most importantly, the lack of improvement in mental health outcomes after a short workshop-program brings concern, knowing that these students might graduate with an ongoing impaired mental health status and move on to a professional career without a safety net provided by the professional health service found within the educational system. Although dancing involves repetitive high-impact movements and a high training load, both of which may explain the high risk of injuries, the dance-specific culture which encouraged the idealization of being lean may also be essential reasons for the high frequency of injuries in dancers [4]. Indeed, we recently found that both LEA and progression in the academic year increase the risk of having an injury by the odds ratio of 1.33 and 2.86, respectively [22]. The culture of high achievement and idealization of leanness in dance, and the prevalence of injuries, may be influential reasons for the high frequency of mental health challenges in professional dance students [22]. This is important information for the physical rehabilitation service within such practice, as dancers are frequently consulting with physical therapists [42], and still possess low mental health literacy [10], and pursue less frequent support from mental health specialists [12].

### Knowledge on sports nutrition and recovery strategies

This intervention was successful at increasing the nutritional knowledge among dancers, with small to moderate effects and with no significant difference from the reference group. This may imply that the detailed nutritional awareness needs repeating or given more continuous focus to manifest and sustain increased nutritional knowledge. While the intervention was designed predominantly to improve mental health literacy, it also included specific content on the consequences of idealizing body figure (e.g., low energy availability), as well as knowledge regarding sports nutrition and recovery strategies. All of these factors are equally important in understanding the underlying mental health states of dancers. Each of these specific elements may have had too narrow a focus, or the time between each workshop may have been too long to be able to build on the previous learning outcomes, and as such, significant knowledge improvements faltered. However, the awareness of nutrition, as an important component of recovery from exercise, was significantly better among dancers compared to references. While scoring for nutrition as a recovery strategy did not improve within the group of dancers, the high scoring noted could indicate positive behavioral outcomes if combined with increased nutritional knowledge. Specifically, the improvements in mental health and nutrition knowledge and reduction in driven exercise behavior may have positive implications with regard to the high incidence of symptoms of LEA and injuries previously reported from this athletic sample [22]. The lesson learned from this intervention is that its focus and content are highly appreciated by the dance students but that they require more comprehensive information about each specific content area, with special emphasis on nutrition. As such, it would be advantageous to implement more routine programs for addressing the long-held culture of normalizing pain and physical and mental exhaustion and body figure idealization, thus allowing for a greater comprehensive learning content.

### The effect of COVID-19 on mental health and behavior in dance students

Most dance students did not report any issues related to COVID-19, although the total hours of exercise increased. The finding of increased exercise in dance students contrasts with the general population, in which physical activity typically decreased [48]. The knowledge that physical activity may serve as a coping strategy for stress may explain our findings about one-third reported coping impairments, with experiences of increased anxiety and depression, concerns about body figure, food intake, and/or energy needs, and distress regarding their future careers. Supporting the suggestion of a potential relationship between the mental stress from COVID-19 and increased level of physical activity is the lack of intervention effect on driven exercise at follow-up.

### Limitations

Dropout rate and the lack of a comparative group during follow-up make the interpretation of differences between groups, and true outcomes from the intervention, challenging to conclude. This can be observed when evaluating the follow-up findings on mental health (e.g., symptoms of eating disorders, symptoms of LEA), as COVID-19 might have confounded these results that otherwise could have improved with time. Additionally, relying on self-reports, and not including any physical examination or clinical interview, must be taken into consideration when evaluating the results of this study. Inviting the full sample of professional dance students in Norway, with the majority consenting to participate, justifies the findings as generalizable to at least the Scandinavian population. However, the lack of ethnic diversity and the number of missing responses from post-intervention and follow-up measures imply the need for precaution when interpreting the findings. Furthermore, not evaluating each workshop individually may obscure the potential for incremental improvements. Nevertheless, the open evaluation question encouraged more detailed elaboration, and this feedback indicated the need for more in-depth knowledge on each of the workshop topics. As such, the evaluations confirmed our presumption of the need for a continuous educational program to provide for more comprehensive education. Finally, a multi-topic intervention covered in a few workshops may have provided a too short time and opportunity to explore the effectiveness one may achieve by promoting dissonant thinking processes. As such, we were able to increase knowledge from participating in interactive workshops, but the lack of change in behavior and mental health symptoms may reasonably be due to the lack of repetition with the dissonance exposure exercises within each topic.

## Conclusion

This short intervention resulted in a temporary reduction in the driven exercise behavior of dance students, and sustained improvements in mental health and nutritional knowledge. The lack of improvements in other domains covered by this intervention suggested the need for a more continuous educational program to ensure acceptance of and personal implementation of the topics. The lack of repeated dissonance cognition exercises related to each of the topics (i.e., body figure idealization, mental health, and sports nutrition) may also explain some of the inconclusive results. This is specifically relevant considering the long-held problematic culture within the art of dance, idealizing a lean body figure at the cost of health, transferred by older students and by pedagogues to younger students [5]. The student's requests for a more individualized approach should also be taken into consideration.

## Data availability statement

The raw data supporting the conclusions of this article will be made available by the authors, without undue reservation.

## Ethics statement

The studies involving human participants were reviewed and approved by Norwegian Regional Committee for Medical and Health Research Ethics (ID 11472). The patients/participants provided their written informed consent to participate in this study.

## Author contributions

JS-B and BA designed the intervention with guidance and feedback from TM. BA managed logistics and communication with the participants during the project period. JS-B and BA held the intervention workshops. TM recruited control group participants, did statistics, and drafted the manuscript. CS-B performed interviews with the participants. All authors contributed to the analyses and manuscript writing. All authors contributed to the article and approved the submitted version.

## Funding

We are grateful for the financial support from the volunteer organization The Norwegian Association of Youth Mental Health and the Dam Foundation.

## Conflict of interest

The authors declare that the research was conducted in the absence of any commercial or financial relationships that could be construed as a potential conflict of interest.

## Publisher's note

All claims expressed in this article are solely those of the authors and do not necessarily represent those of their affiliated organizations, or those of the publisher, the editors and the reviewers. Any product that may be evaluated in this article, or claim that may be made by its manufacturer, is not guaranteed or endorsed by the publisher.
