# Supplementary material for: Intervention in professional dance students to increase mental health- and nutrition literacy: A controlled trial with follow up
Source: Front Sports Act Living. 2022 Sep 21;4:727048. doi: 10.3389/fspor.2022.727048 (PMC9532567; doi:10.3389/fspor.2022.727048)
Supplement: Supplementary file 1 [file Data_Sheet_1.PDF]

Supplementary Material

Supplementary Table 1: Workshops I-III in the *Choreography of the mind* intervention

**Workshop I**<br>**Mental health part 1**<br>**90 minutes**		
MAIN CONTENT	TECHNIQUE	TARGETS
**What is mental health?**<br>o Definition of mental health, mental distress, and mental illness<br>o Prevalence of mental distress and illness	Theoretical presentation by workshop presenter	Increase awareness and knowledge of mental health concepts
**The importance of focusing on mental health among dancers**<br>o Health, performance, motivation, quality of life, and daily function perspectives	Interactive presentation by workshop presenter with the opportunity for students to exemplify and comment.	Increase the understanding of why mental health is important within dance and for them as dance students individually
**Common mental health challenges**<br>o Symptoms of mental health issues in general<br>o Anxiety, depression, obsessive compulsive disorders, perfectionism<br>• Symptoms and how to recognize<br>o Eating disorders<br>• What is it, and who are affected?<br>• The different types of eating disorders<br>• Consequences (bone health, relative energy deficiency in sports (RED-s), immune system)<br>• Symptoms (physiological, psychological, behavioral)	Theoretical presentation by workshop presenter	Develop an awareness and knowledge of specific issues and challenges that are prevalent within dancers' environment, and how to recognize issues among other students and within themselves
**How can each individual dancer contribute to the promotion of good mental health?**<br>o For oneself	Discussion among students and in plenary	Internalize an understanding of how they should and can contribute to

o For others		promote mental health within their environment
**Workshop II**<br>**Mental health part 2 & Sports nutrition**<br>**90 minutes**		
MAIN CONTENT	TECHNIQUE	TARGETS
**Repetition from mental health part I**<br>o Symptoms of mental health issues and eating disorders	Theoretical presentation by workshop presenter	Repeat the most important information to enhance learning
**Mental health part II**<br>o When do mental health issues become a problem?<br>o What issues can be considered normal?<br>o When and where can you seek help?	Interactive presentation by workshop presenter with the opportunity for students to exemplify and comment	Improve awareness and ability to recognize issues to prevent sever challenges or illness to evolve/develop.
**Relative energy deficiency in sports (RED-s)**<br>o What is RED-s?<br>o Consequences<br>o «The traffic light» concept used by the Norwegian Olympic Sports Centre	Theoretical presentation by workshop presenter	Develop knowledge related to the topic to better understand the risks and consequences of RED-s
**Sports nutrition**<br>o The dietary circle and dietary pyramid<br>o The meal plate for different exercise intensities and volume<br>o What is it, why do we need them, and where to get them?<br>• Macronutrients<br>• Micronutrients<br>o Dietary supplements<br>o Meal frequency<br>o Energy availability, RED-s and bone health<br>o Tricks on how to consume enough energy<br>o Energy and fluid needs related to 1. practice and 2. performance/show<br>• Pre-, during, and post-practice/performance<br>• What, when and why?	Interactive presentation by workshop presenter with the opportunity for students to exemplify and comment.	Develop knowledge to internalize the importance to them as dancers, to establish tools on how to manage in a busy schedule, and to further prevent destructive dietary habits and promote healthy and performance improving attitudes and habits within the dancers' environment and among themselves.

• Practical solutions<br>o Recovery<br>• Diet, fluid, rest, sleep, recovery exercise, stress		
**Workshop III**<br>**Role play: how to deal with mental health challenges**<br>**90 minutes**		
MAIN CONTENT	TECHNIQUE	TARGETS
**Role play**<br><br>***<u>Case 1</u>***<br>One of your dance students has changes his behavior since last fall. He looks sad, seems to lack motivation, and is less social.<br><br>Create a role play where one dance student plays this male dancer, and the other dance students are trying to get this dancer to start talking and maybe recommend him to seek help to deal with his challenges<br><br>***<u>Case 2</u>***<br>One of the dance students continuously talks about how dissatisfied she is with herself, that she feels fat, and is too big to be a dancer.<br><br>Create a role play where one dance student is this female dancer, and where two other dance students are communication with her. The purpose is to focus on how you as a group can reduce the negative body focus.<br><br>***<u>Case 3</u>***<br>Your school is visited by young promising dancers who are considering applying to this school next semester. You are given the task to hold a presentation related to sports nutrition, and the need for healthy dietary habits as a dancer.	Interactive learning through case solving and role play<br><br>Students are divided into groups. Each group are given different cases to solve, and to present to the rest of the groups. All cases and role play solutions are discussed after presentations.	Improve the dancers' ability to communicate on these topics, and to lower the bar on how to help others and to seek help themselves.

Prepare a 10-minute presentation.<br><br>***<u>Case 4</u>***<br>One of the dance teachers tends to regularly use a language where there is a focus on body appearance. You also know that some of the dance students in your class are negatively affected by this behavior.<br><br>Create a role play where you plan a conversation with this teacher.<br>After this, one of the students play the teacher who the other students then have this planned conversation with. The purpose of this conversation is to make the teacher aware of her use of words and body language, and its negative affect on her dance students.<br><br>***<u>Case 5</u>***<br>Imagine that one of the female dancers in your class has lost some weight (visible for you) and only eats specific vegetables and non-fat, low carbohydrate crackers or toast, most often without topping, during the day. She appears to be tiered and lack energy.<br><br>Create a role play where one of the students play this female dance student, and where the other students are trying to make her talk about how she really feels.<br><br>***<u>Case 6</u>***<br>Imagine a role play where one of you as a student, has challenges related to anxiety/depression. The one who experience challenges has now sought help from one of the other students to talk about his/her challenges. How does the dialogue play out, and how does it end?		

Supplementary Table 2: Mental health Scores were from 0-3 for each of the questions.

	**How do you define mental health**	**When does an everyday problem become a mental health symptom**	**Give 1-3 examples of physical symptoms on mental health issues**	**Give 1-3 examples of psychological symptoms on mental health issues**
Scores	0-3	0-3	0-3	0-3
Explanation	*Higher scores if giving more advanced explanations*	*Higher scores if giving more advanced explanations*	*One point for each valid example*	*One point for each valid example*

3 **Supplementary Table 3:** An overview on the evaluation of dietary knowledge. Food items qualifying for scores, and food items typically reported that were not qualifying for scores, are presented. For macronutrients, participants were given a score from 0 to 4 if being able to name up to four food groups for each nutrient, while for micronutrients the scores were from 0-3

**Nutrient**	**Food items qualifying**	**Food items not qualifying**
Carbohydrate	Bread/cereals/grains, rice, pasta, legumes, fruits, quinoa	Crisp bread, vegetables, nuts
Proteins	Meat, fish, dairy products, egg, tofu, soy-products, legumes	Nuts
Unsaturated fats	Vegetable oils, avocado/olives, nuts/seeds, margarine	Dairy products/butter, egg, meat, coconut
Calcium	Dairy products, green leafy vegetables, almonds	Vegetables in general, nuts
Iron	Meat, egg, liver(pate), bread/cereal/grains, brown cheese	Green vegetables, cocoa, nuts
